# The Annual American Men's Internet Survey of Behaviors of Men Who have Sex with Men in the United States: 2014 Key Indicators Report

**DOI:** 10.2196/publichealth.5476

**Published:** 2016-05-25

**Authors:** Travis Sanchez, Maria Zlotorzynska, Craig Sineath, Erin Kahle, Patrick Sullivan

**Affiliations:** ^1^Emory UniversityRollins School of Public HealthAtlanta, GAUnited States

**Keywords:** MSM, gay, homosexual, bisexual, HIV, STD, Internet, survey, surveillance

## Abstract

The American Men’s Internet Survey (AMIS) is an annual Web-based behavioral survey of men who have sex with men (MSM) who live in the United States. The purpose of this Rapid Surveillance Report is to report on the second cycle of data collection (November 2014 through April 2015; AMIS-2014) on the same key indicators previously reported for AMIS (December 2013 through May 2014; AMIS-2013). The AMIS survey methodology has not substantively changed since AMIS-2013. MSM were recruited from a variety of websites using banner advertisements or email blasts. Adult men currently residing in the United States were eligible to participate if they had ever had sex with a man. We examined demographic and recruitment characteristics using multivariable regression modeling (*P*<.05) stratified by the participants' self-reported human immunodeficiency virus (HIV) status. The AMIS-2014 round of data collection resulted in 9248 completed surveys from MSM representing every US state. Participants were mainly white, 40 years or older, living in the US South, living in urban/suburban areas, and recruited from a general social networking website. Self-reported HIV prevalence was 11.34% (1049/9248).

Compared with HIV-negative/unknown status participants, HIV-positive participants were more likely to have had anal sex without a condom with any male partner in the past 12 months (76.55% vs 67.17%; *P*<.001) and more likely to have had anal sex without a condom with their last male sex partner who was discordant/unknown HIV status (39.66% vs 18.77%; *P*<.001). Marijuana and other illicit substance use in the past 12 months was more likely to be reported by HIV-positive participants than HIV-negative/unknown status participants (26.02% vs 21.27%, and 27.26% vs 17.60%, respectively; both *P*<.001). The vast majority (86.90%, 7127/8199) of HIV-negative/unknown status participants had been previously HIV tested, and 58.23% (4799/8199) had been tested in the past 12 months. Sexually transmitted infection (STI) testing and diagnosis was also more likely to be reported by HIV-positive participants than HIV-negative/unknown status participants (71.02% vs 37.34%, and 20.59% vs 7.54%, respectively; both *P*<.001). HIV-negative/unknown status participants <40 years of age were more likely than those 40 years or older to have had anal sex without a condom, were more likely to report substance use, were less likely to have been HIV tested, but were more likely to been tested for and diagnosed with an STI. Compared with those from general social networking, HIV-negative/unknown status participants from a geospatial social networking website were more likely to have reported all risk behaviors but were more likely to have been HIV tested, STI tested, and diagnosed with an STI.

Notice to the reader: Rapid Surveillance Reports are brief reports, which primarily report new data in table format from an existing well-described surveillance system, making a methods (and sometimes an introduction) section redundant. The idea of this new article type is to allow rapid publication of emerging trends, or continuous publication in regular intervals of public health relevant data. If a method or system description has been published previously in JMIR Public Health Surveill or JMIR Res Protoc, the report does not have to be peer-reviewed again (although in many cases they still are).

## Introduction

The American Men’s Internet Survey (AMIS) is an annual Web-based behavioral survey of men who have sex with men who live in the United States. The methods have been previously published [1]. Methods in AMIS-2014 are unchanged from the previously published manuscript unless otherwise noted below.

### Recruitment and Enrollment

As in the prior year, AMIS participants were recruited through convenience sampling from a variety of websites using banner advertisements or email blasts to website members (hereafter referred to generically as “ads”). Men who clicked on the ads were taken directly to the survey website hosted on a secure server administered by SurveyGizmo. To be eligible for the survey, participants had to be 15 years of age or older, consider themselves to be male, reside in the United States, and report that they had oral or anal sex with a man at least once in the past (hereafter referred to as MSM). Persons who reported being <15 years of age or refused to provide their age were not asked any other screening questions. MSM who met the eligibility criteria and consented to participate in the study started the Web-based survey immediately. The full questionnaire for AMIS-2014 is presented in Multimedia Appendix 1. AMIS-2014 ran from November 2014 through April 2015, and resulted in 77,611 persons clicking on the ads and landing on the study's recruitment page (Table 1). Most were from a general social networking website (59,670/77,611, 76.88%). Nearly half (35,462/77,611 46.89%) of those who landed on the study's page started the screening process and 60.75% (47,149/77,611) were eligible. The most common reason for ineligibility was not ever having male-male sex. Nearly three-quarters (57,176/77,611, 73.67%) of those who were eligible consented to participate in the survey. There were 6.81% (1109/77,611) of the surveys determined to likely be from duplicate participants. Among unduplicated surveys, more than two-thirds (52,9790/77,611, 68.25%) were considered successful. Success was defined using an examination of completed survey sections [1]. Most successful surveys were among men who reported having sex with another man in the past 12 months (9248/10,359, 89.28%).

### Measures and Analyses

For AMIS-2014 analyses, we categorized participants by recruitment website and based on target audience and purpose: gay social networking (n=2), gay general interest (n=4), general social networking (n=1), and geospatial social networking (n=2). We do not provide the names of the websites to preserve operator/client privacy, particularly where a website category has only one operator. The participants who were eligible, consented, unduplicated, successful, and reported male-male sex in the past 12 months were included in analyses of participant characteristics and behavior.

The following behavioral measures differed in AMIS-2014 from those previously published: both sexual behaviors (any condomless anal sex and condomless anal sex with a discordant/unknown status partner) were assessed for the past 12 months, binge alcohol drinking was not included, and substance using behaviors were recategorized. Human immunodeficiency virus (HIV) serostatus concordance was based on the participant’s HIV status and the status of their sex partner. Discordant/unknown status was defined as either the participant or partner having unknown status or when one was HIV-negative and the other was HIV-positive. For substance-using behaviors in the past 12 months, we separated marijuana use from other illicit substance use. For AMIS-2014 all participants received questions on sexually transmitted infection (STI; chlamydia, gonorrhea, and syphilis) testing and diagnoses in the past 12 months. Participants could have been tested for an STI but not diagnosed with an STI. Persons who were diagnosed with an STI in the past 12 months all were considered to have been tested for an STI in the past 12 months.

The analysis methods for AMIS-2014 did not substantively differ from those previously published but are repeated in this report for clarity [1]. Overall chi-square tests were used to identify whether participant characteristics significantly differed between recruitment website types. Multivariable logistic regression modeling was used to determine significant differences in behaviors based on self-reported HIV status while controlling for race/ethnicity, age group, National HIV Behavioral Surveillance (NHBS) city residency, and recruitment website type. HIV-testing behaviors were only examined among those who did not report that they were HIV-positive and were also presented by participant characteristics. Multivariable logistic regression results are presented as Wald chi-square *P*-values to denote an independently significant difference in the behavior for each subgroup compared with a referent group. Statistical significance was determined at *P*<.05.

## Results

### Summary for AMIS-2014

**Figure 1:**
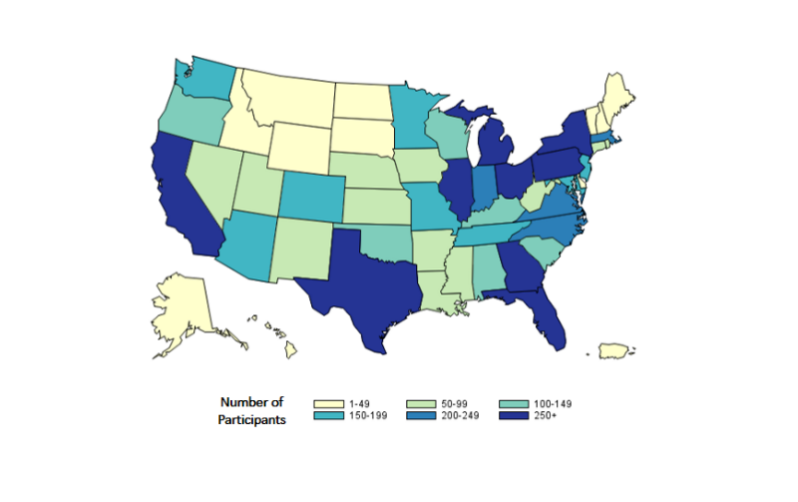
Number of MSM participants in the American Men's Internet Survey by state, 2014.

Three-quarters (6819/9248, 73.73%) of participants included in this report were white, non-Hispanic, half (4676/9248, 50.6%) were ≥40 years of age, and their most common region of residence was the South followed by the West (Table 2). AMIS-2014 had participants from all US states and at least 100 participants from each of 27 states (Figure 1). Overall, 11.34% (1049/9248) of participants reported being HIV positive and 88.66% (8199/9248) reported being HIV negative or having an unknown HIV serostatus. There were significant differences in all participant characteristics based on where they were recruited (Table 2). Most of those differences were observed among participants recruited from geospatial social networking websites, who were less likely be white, less likely be 40 years or older, less likely to live in an NHBS city, more likely to live in the West, more likely to live in urban areas, and more likely to report being HIV positive.

Most participants had anal sex without a condom with another man in the past 12 months (Table 3). Compared with HIV-negative/unknown status participants, those who were HIV-positive were significantly more likely to report anal intercourse without a condom, including with male partners who were discordant/unknown status. Anal intercourse without a condom significantly differed by age group (HIV-positive and -negative/unknown status participants), recruitment website type (HIV-positive and -negative/unknown status participants), and race/ethnicity (HIV-negative/unknown status participants only).

More than one-quarter (273/1049, 26.02%) of HIV-positive participants reported using marijuana or other illicit substances in the past 12 months (Table 4). Compared with HIV-negative/unknown status participants, those who were HIV-positive were significantly more likely to report use of marijuana and other substances in the past 12 months. Marijuana or other illicit substance use significantly differed by age group (HIV-positive and -negative/unknown status participants), residence in an NHBS city (HIV-negative/unknown status participants only), and recruitment website type (HIV-negative/unknown status participants only).

HIV testing behaviors were only examined among those who did not report being HIV-positive. Most of those participants (7125/8199, 86.90%) had ever been previously tested for HIV infection, and just over half (4799/8199, 58.53%) reported being tested in the past 12 months (Table 5). HIV testing significantly differed by age group (ever tested), race/ethnicity (ever tested), residence in an NHBS city (past 12 months tested), and recruitment website type (past 12 months tested).

Compared with HIV-negative/unknown status participants, those who were HIV-positive were more likely to report being tested for and diagnosed with an STI in the past 12 months (Table 6). The most common STI diagnoses were syphilis (132/1049, 12.58%) and chlamydia (88/1049, 8.39%) among HIV-positive participants. STI testing significantly differed by age group, residence in an NHBS city and recruitment website type only for participants who were HIV-negative/unknown status. STI diagnosis significantly differed by age group (HIV-positive and HIV-negative/unknown status participants), race/ethnicity (HIV-negative/unknown status participants only), residence in an NHBS city (HIV-negative/unknown status participants only), and recruitment website type (HIV-negative/unknown status participants only).

**Table 1:** Recruitment outcomes with different recruitment website types for the American Men's Internet Survey, United States, 2014.

				Recruitment Website Type							
		Total		Gay social networking (n=2)		General gay interest (n=4)		General social networking (n=1)		Geospatial social networking (n=2)	
Recruitment Outcomes		N	(%)	N	(%)	N	(%)	N	(%)	N	(%)
Clicked ad		77,611		1988		8372		59,670		7581	
Screened^a^		36,392	(46.89)	944	(47.48)	1293	(15.44)	26,576	(44.54)	7579	(99.97)
Ineligible^b^		14,285	(39.25)	171	(18.11)	523	(40.45)	11,356	(42.73)	2235	(29.49)
	Not 15+ years of age^c^	10,219	(71.54)	118	(69.01)	340	(65.01)	8008	(70.52)	1753	(78.43)
	Not male^c^	10,942	(76.60)	138	(80.70)	380	(72.66)	8494	(74.80)	1930	(86.35)
	Not ever MSM^c,d^	13,776	(96.44)	167	(97.66)	510	(97.51)	11,073	(97.51)	2026	(90.65)
	Not a US resident^c^	3733	(26.13)	22	(12.87)	161	(30.78)	2460	(21.66)	1090	(48.77)
Eligible^b^		22,107	(60.75)	773	(81.89)	770	(59.55)	15,220	(57.27)	5344	(70.51)
Consented^e^		16,286	(73.67)	574	(74.26)	595	(77.27)	10,821	(71.10)	4296	(80.39)
Unduplicated^f^		15,177	(93.19)	557	(97.04)	564	(94.79)	9960	(92.04)	4096	(95.34)
Success^g^		10,359	(68.25)	414	(74.33)	410	(72.70)	6913	(69.41)	2622	(64.01)
MSM past 12 months^h^		9248	(89.28)	377	(91.06)	369	(90.00)	5987	(86.60)	2515	(95.92)

^a^Proportion is of total who clicked ad. Includes those who started the screening questionnaire.

^b^Proportion is among total screened. Ineligible includes those who did not complete the screening questionnaire.

^c^Proportion is among total ineligible. Includes those who may not have responded to the question.

^d^MSM: men who have sex with men.

^e^Proportion is among eligible.

^f^Proportion is among consented. Unduplicated removes participants who were marked as duplicates using an Internet protocol address and demographic data matching.

^g^Proportion is among unduplicated. Success removes participants who did not pass the test for survey completeness.

^h^Proportion is among successes.

**Table 2:** Characteristics of MSM participants in the American Men's Internet Survey by recruitment website type, United States, 2014.

				Recruitment website type								
Participant characteristics		Total		Gay social networking (n=2)		General gay interest (n=4)		General social networking (n=1)		Geospatial social networking (n=2)		
		N	(%)	N	(%)	N	(%)	N	(%)	N	(%)	*P* -value^a^
Race/ethnicity												<.001
	Black, non-Hispanic	415	(4.49)	11	(2.92)	21	(5.69)	225	(3.76)	158	(6.28)	
	Hispanic^b^	1308	(14.14)	14	(3.71)	37	(10.03)	713	(11.91)	544	(21.63)	
	White, non-Hispanic	6819	(73.73)	327	(86.74)	286	(77.51)	4643	(77.55)	1563	(62.15)	
	Other or multiple races	706	(7.63)	25	(6.63)	25	(6.78)	406	(6.78)	250	(9.94)	
Age (years)												<.001
	15-24	1389	(15.02)	21	(5.57)	47	(12.74)	857	(14.31)	464	(18.45)	
	25-29	1221	(13.20)	24	(6.37)	48	(13.01)	612	(10.22)	537	(21.35)	
	30-39	1962	(21.22)	40	(10.61)	86	(23.31)	1164	(19.44)	672	(26.72)	
	40 or older	4676	(50.56)	292	(77.45)	188	(50.95)	3354	(56.02)	842	(33.48)	
Region												<.001
	Midwest	1560	(16.87)	68	(18.04)	64	(17.34)	985	(16.45)	443	(17.61)	
	Northeast	1933	(20.90)	117	(31.03)	55	(14.91)	1306	(21.81)	455	(18.09)	
	South	3634	(39.29)	122	(32.36)	180	(48.78)	2369	(39.57)	963	(38.29)	
	West	2110	(22.82)	69	(18.30)	65	(17.62)	1323	(22.10)	653	(25.96)	
	US dependent areas	11	(0.12)	1	(0.27)	5	(1.36)	4	(0.07)	1	(0.04)	
NHBS city resident^c^												<.001
	Yes	3553	(38.42)	137	(36.34)	206	(55.83)	2177	(36.36)	1033	(41.07)	
	No	5695	(61.58)	240	(63.66)	163	(44.17)	3810	(63.64)	1482	(58.93)	
Population density^d^												<.001
	Rural	2774	(30.00)	133	(35.28)	80	(21.68)	1902	(31.77)	659	(26.20)	
	Urban/suburban	6300	(68.12)	241	(63.93)	285	(77.24)	4001	(66.83)	1773	(70.50)	
Self-reported HIV status												<.001
	Positive	1049	(11.34)	23	(6.10)	32	(8.67)	554	(9.25)	440	(17.50)	
	Negative	6992	(75.61)	277	(73.47)	303	(82.11)	4588	(76.63)	1824	(72.52)	
	Unknown	1207	(13.05)	77	(20.42)	34	(9.21)	845	(14.11)	251	(9.98)	
Total		9248		377		369		5987		2515		

^a^Chi-square testing difference in characteristics between website type.

^b^Hispanic persons could have been of any race, including other or multiple.

^c^NHBS: National HIV Behavioral Surveillance System.

^d^There were 71 participants missing information needed to determine the population density of the area where they lived.

**Table 3:** Sexual behaviors with male partners of MSM participants in the American Men's Internet Survey, United States, 2014.

			Sexual Behaviors with male partners in the past 12 months					
Participant characteristics		N in sample	Anal intercourse without a condom			Anal intercourse without a condom with a partner of discordant or unknown HIV status			
			n	(%)	*P* -value^a^	n	(%)	*P* -value^a^
**HIV positive overall**		**1049**	**803**	**(76.55)**	**<.001**^b^	**416**	**(39.66)**	**<.001**^b^
Race/ethnicity								
	Black, non-Hispanic	92	69	(75.00)	.459	34	(36.96)	.253
	Hispanic	172	134	(77.91)	.636	73	(42.44)	.893
	White, non-Hispanic	716	546	(76.26)	REF	281	(39.25)	REF
	Other or multiple races	69	54	(78.26)	.878	28	(40.58)	.794
Age (years)									
	15-24	68	62	(91.18)	.038	38	(55.88)	.090
	25-29	110	96	(87.27)	.226	65	(59.09)	.002
	30-39	251	199	(79.28)	.086	111	(44.22)	.278
	40 or older	620	446	(71.94)	REF	202	(32.58)	REF
NHBS city resident^c^								
	Yes	464	361	(77.80)	.217	173	(37.28)	.291
	No	585	442	(75.56)	REF	243	(41.54)	REF
Recruitment website type								
	Gay social networking	23	18	(78.26)	.620	16	(69.57)	.007
	General gay interest	32	24	(75.00)	.768	17	(53.13)	.478
	General social networking	554	403	(72.74)	REF	192	(34.66)	REF
	Geospatial social networking	440	358	(81.36)	.642	191	(43.41)	.002
**HIV negative or unknown overall**		**8199**	**5507**	**(67.17)**	**REF**	**1539**	**(18.77)**	**REF**
Race/ethnicity								
	Black, non-Hispanic	323	202	(62.54)	.043	80	(24.77)	.121
	Hispanic	1136	777	(68.40)	.377	276	(24.30)	.088
	White, non-Hispanic	6103	4109	(67.33)	REF	1053	(17.25)	REF
	Other or multiple races	637	419	(65.78)	.808	130	(20.41)	.323
Age (years)									
	15-24	1321	870	(65.86)	<.001	334	(25.28)	.359
	25-29	1111	820	(73.81)	<.001	193	(17.37)	.954
	30-39	1711	1281	(74.87)	<.001	241	(14.09)	.572
	40 or older	4056	2536	(62.52)	REF	505	(12.45)	REF
NHBS city resident^c^									
	Yes	3089	2053	(66.46)	.300	607	(19.65)	.442
	No	5110	3454	(67.59)	REF	932	(18.24)	REF
Recruitment website type									
	Gay social networking	354	187	(52.82)	<.001	76	(21.47)	.318
	General gay interest	337	219	(64.99)	.909	68	(20.18)	.680
	General social networking	5433	3592	(66.11)	REF	825	(15.18)	REF
	Geospatial social networking	2075	1509	(72.72)	<.001	570	(27.47)	<.001

^a^Wald chi-square from multivariable logistic regression comparing behavior (yes versus no) among group with some characteristic compared to a referent (REF) group.

^b^Wald chi-square from multivariable logistic regression comparing behavior (yes versus no) among HIV-positive participants compared to HIV-negative or unknown serostatus participants. Model controlled for race/ethnicity, age, NHBS residency, and website type.

^c^NHBS: National HIV Behavioral Surveillance System.

**Table 4:** Substance using behaviors of MSM participants in the American Men's Internet Survey, United States, 2014.

			Substance use behaviors in the past 12 months					
Participant characteristics		N in sample	Used marijuana			Used other substance(s)		
			n	(%)	*P* -value^a^	N	(%)	*P* -value^a^
**HIV positive overall**		**1049**	**273**	**(26.02)**	**<.001**^b^	**286**	**(27.26)**	**<.001**^b^
Race/ethnicity								
	Black non-Hispanic	92	24	(26.09)	.549	19	(20.65)	.110
	Hispanic	172	46	(26.74)	.468	44	(25.58)	.571
	White non-Hispanic	716	181	(25.28)	REF	202	(28.21)	REF
	Other or multiple races	69	22	(31.88)	.359	21	(30.43)	.400
Age (years)								
	15-24	68	21	(30.88)	.988	17	(25.00)	.360
	25-29	110	40	(36.36)	.082	34	(30.91)	.441
	30-39	251	76	(30.28)	.771	91	(36.25)	.011
	40 or older	620	136	(21.94)	REF	144	(23.23)	REF
NHBS city resident^c^								
	Yes	464	123	(26.51)	.781	130	(28.02)	.573
	No	585	150	(25.64)	REF	156	(26.67)	REF
Recruitment website type								
	Gay social networking	23	7	(30.43)	.588	4	(17.39)	.316
	General gay interest	32	10	(31.25)	.610	11	(34.38)	.255
	General social networking	554	119	(21.48)	REF	134	(24.19)	REF
	Geospatial social networking	440	137	(31.14)	.971	137	(31.14)	.353
**HIV negative or unknown overall**		**8199**	**1744**	**(21.27)**	**REF**	**1443**	**(17.60)**	**REF**
Race/Ethnicity								
	Black non-Hispanic	323	60	(18.58)	.051	50	(15.48)	.060
	Hispanic	1136	256	(22.54)	.586	212	(18.66)	.596
	White non-Hispanic	6103	1274	(20.87)	REF	1051	(17.22)	REF
	Other or multiple races	637	154	(24.18)	.123	130	(20.41)	.102
Age (years)								
	15-24	1321	424	(32.10)	<.001	269	(20.36)	.160
	25-29	1111	303	(27.27)	.005	259	(23.31)	.002
	30-39	1711	411	(24.02)	.912	382	(22.33)	.002
	40 or older	4056	606	(14.94)	REF	533	(13.14)	REF
NHBS city resident^c^								
	Yes	3089	717	(23.21)	<.001	656	(21.24)	<.001
	No	5110	1027	(20.10)	REF	787	(15.40)	REF
Recruitment website type								
	Gay social networking	354	48	(13.56)	.032	39	(11.02)	.022
	General gay interest	337	69	(20.47)	.985	55	(16.32)	.535
	General social networking	5433	1095	(20.15)	REF	861	(15.85)	REF
	Geospatial social networking	2075	532	(25.64)	<.001	488	(23.52)	<.001

^a^Wald chi-square from multivariable logistic regression comparing behavior (yes versus no) among group with some characteristic compared with a referent (REF) group.

^b^Wald chi-square from multivariable logistic regression comparing behavior (yes versus no) among HIV-positive participants compared to HIV-negative or unknown serostatus participants. Model controlled for race/ethnicity, age, NHBS residency, and website type.

^c^NHBS: National HIV Behavioral Surveillance System.

**Table 5:** HIV testing behaviors of HIV-negative or unknown status MSM participants in the American Men's Internet Survey, United States, 2014.

		HIV testing behaviors						
Participant characteristics		N in sample	HIV tested ever			HIV tested past 12 months		
			n	(%)	*P* -value^a^	N	(%)	*P* -value^a^
Race/ethnicity								
	Black non-Hispanic	323	287	(88.85)	.503	213	(65.94)	.221
	Hispanic	1136	981	(86.36)	.544	706	(62.15)	.290
	White non-Hispanic	6103	5299	(86.83)	REF	3454	(56.60)	REF
	Other or multiple races	637	558	(87.60)	.469	406	(63.74)	.308
Age (years)								
	15-24	1321	892	(67.52)	<.001	699	(52.91)	<.001
	25-29	1111	997	(89.74)	.006	742	(66.79)	<.001
	30-39	1711	1547	(90.41)	<.001	1037	(60.61)	.498
	40 or older	4056	3689	(90.95)	REF	2301	(56.73)	REF
NHBS city resident^b^								
	Yes	3089	2787	(90.22)	<.001	1992	(64.49)	<.001
	No	5110	4338	(84.89)	REF	2787	(54.54)	REF
Recruitment website type								
	Gay social networking	354	280	(79.10)	<.001	172	(48.59)	<.001
	General gay interest	337	304	(90.21)	.054	188	(55.79)	.068
	General social networking	5433	4662	(85.81)	REF	2881	(53.03)	REF
	Geospatial social networking	2075	1879	(90.55)	<.001	1538	(74.12)	<.001
TOTAL		8199	7125	(86.90)		4799	(58.53)	

^a^Wald chi-square from multivariable logistic regression comparing behavior (yes versus no) among group with some characteristic compared with a referent (REF) group.

^b^NHBS: National HIV Behavioral Surveillance System.

**Table 6:** Sexually transmitted infection testing and diagnosis of MSM participants in the American Men's Internet Survey, United States, 2014.

			STI History in the past 12 months					
Participant characteristics		N in sample	Tested for any STI^a^			Diagnosed with any STI^a^		
			n	(%)	*P* -value^b^	N	(%)	*P* -value^b^
**HIV positive overall**		**1049**	**747**	**(71.21)**	**<.001**^c^	**216**	**(20.59)**	**<.001**^c^
Race/ethnicity								
	Black non-Hispanic	92	72	(78.26)	.508	25	(27.17)	.905
	Hispanic	172	129	(75.00)	.354	47	(27.33)	.687
	White non-Hispanic	716	493	(68.85)	REF	120	(16.76)	REF
	Other or multiple races	69	53	(76.81)	.867	24	(34.78)	.065
Age (years)								
	15-24	68	57	(83.82)	.365	20	(29.41)	.968
	25-29	110	94	(85.45)	.086	44	(40.00)	<.001
	30-39	251	203	(80.88)	.583	67	(26.69)	.846
	40 or older	620	393	(63.39)	REF	85	(13.71)	REF
NHBS city resident^d^								
	Yes	464	344	(74.14)	.073	105	(22.63)	.326
	No	585	403	(68.89)	REF	111	(18.97)	REF
Recruitment website type								
	Gay social networking	23	13	(56.52)	.282	2	(8.70)	.302
	General gay interest	32	24	(75.00)	.416	8	(25.00)	.154
	General social networking	554	364	(65.70)	REF	81	(14.62)	REF
	Geospatial social networking	440	346	(78.64)	.214	125	(28.41)	.246
**HIV negative or unknown overall**		**8199**	**3086**	**(37.64)**	**REF**	**618**	**(7.54)**	**REF**
Race/Ethnicity								
	Black non-Hispanic	323	156	(48.30)	.272	35	(10.84)	.559
	Hispanic	1136	553	(48.68)	.173	147	(12.94)	.007
	White non-Hispanic	6103	2086	(34.18)	REF	378	(6.19)	REF
	Other or multiple races	637	291	(45.68)	.470	58	(9.11)	.454
Age (years)								
	15-24	1321	512	(38.76)	.033	109	(8.25)	.612
	25-29	1111	579	(52.12)	<.001	136	(12.24)	.001
	30-39	1711	754	(44.07)	.040	174	(10.17)	.021
	40 or older	4056	1241	(30.60)	REF	199	(4.91)	REF
NHBS city resident^d^								
	Yes	3089	1442	(46.68)	<.001	300	(9.71)	<.001
	No	5110	1644	(32.17)	REF	318	(6.22)	REF
Recruitment website type								
	Gay social networking	354	84	(23.73)	<.001	14	(3.95)	.173
	General gay interest	337	109	(32.34)	.013	17	(5.04)	.107
	General social networking	5433	1740	(32.03)	REF	284	(5.23)	REF
	Geospatial social networking	2075	1153	(55.57)	<.001	303	(14.60)	<.001

^a^STI: sexually transmitted infection; includes chlamydia, gonorrhea and syphilis.

^b^Wald chi-square from multivariable logistic regression comparing behavior (yes versus no) among group with some characteristic compared with a referent (REF) group.

^c^Wald chi-square from multivariable logistic regression comparing behavior (yes versus no) among HIV-positive participants compared with HIV-negative or unknown serostatus participants. Model controlled for race/ethnicity, age, NHBS residency, and website type.

^d^NHBS: National HIV Behavioral Surveillance System.

## Abbreviations

AMIS: American Men’s Internet Survey
HIV: human immunodeficiency virus
MSM: men who have sex with men
NHBS: national HIV behavioral surveillance system
STI: sexually transmitted infection

## Multimedia Appendix 1

American Men's Internet Survey, 2014.
