# Supplementary material for: The Annual American Men's Internet Survey of Behaviors of Men Who have Sex with Men in the United States: 2014 Key Indicators Report
Source: JMIR Public Health Surveill. 2016 May 25;2(1):e23. doi: 10.2196/publichealth.5476 (PMC4909387; doi:10.2196/publichealth.5476)
Supplement: Supplementary file 1 [file publichealth_v2i1e23_app1.pdf]

American Men’s Internet Survey - 2014

Survey Domains **Page Number**

Eligibility...1

Consent...3

Demographics...5

Sexual Behavior with Females in the Past 12 Months...9

Sexual Behavior with Males in the Past 12 Months...12

Sexual Behavior with Last Male Partner...16

Injection Drug Use...23

Non-injection Drug Use...25

Alcohol or Drugs at Last Sex...27

HIV Testing...30

PrEP and nPEP...35

Acute Infection...40

STIs...42

Prevention Activities...47

Join over 10,000 men in the U.S. by taking our annual sexual health survey!

"Sex is the Question" collects information to help researchers better understand patterns of behavior – both sexual and health promoting – among men and to help ensure prevention resources have the greatest impact for the community. Click or tap the arrow or "Next" button below to be a part of this national effort!

How old are you?

____

Since October 2014, did you already complete at least part of Sex is the Question?

( ) No

( ) Yes

( ) I prefer not to answer

( ) Don't know

Do you consider yourself to be Hispanic or Latino?

( ) No

( ) Yes

( ) I prefer not to answer

( ) Don't know

Which racial group or groups do you consider yourself to be in? Check all that apply.

[ ] American Indian or Alaska Native

[ ] Asian

[ ] Black or African American

[ ] Native Hawaiian or Other Pacific Islander

[ ] White

[ ] I prefer not to answer

[ ] Does not apply

[ ] Don't know

What zipcode do you live in?

____

Do you consider yourself to be male, female, or transgender?

( ) Male

( ) Female

( ) Transgender

( ) Don't know

( ) Prefer not to answer

Have you <u>ever</u> had vaginal sex (penis in the vagina) or anal sex (penis in the butt) with a woman?

( ) No

( ) Yes

( ) I prefer not to answer

( ) Don't know

Have you <u>ever</u> had oral sex (mouth on the penis) with a man?

( ) No

( ) Yes

( ) I prefer not to answer

( ) Don't know

Have you <u>ever</u> had anal sex (penis in the butt) with a man?

( ) No

( ) Yes

( ) I prefer not to answer

( ) Don't know

Do you consider yourself to be:

( ) Homosexual or Gay

( ) Heterosexual or Straight

( ) Bisexual

( ) I prefer not to answer

( ) Don't know

Thank you for your interest in our survey. Please note:

1. Your answers are confidential: we don't have any information about who you are beyond the questions you answer.

2. This survey includes some personal questions. You can choose to not answer any questions that make you feel uncomfortable.

3. If you agree to be in this study, you will first be screened to see if you qualify to be in the study. If you qualify, you will be given the opportunity to complete the survey.

If you have any questions or comments you may contact the research staff at sexisthequestion@emory.edu.

Please read the information below about the study, and indicate whether you consent to participate in the study.

( ) I consent to participate in the survey.

( ) I do not consent to participate in the survey.

Thank you for your interest in our survey. Please note:

1. Your answers are confidential: we don't have any information about who you are beyond the questions you answer.

2. This survey includes some personal questions. You can choose to not answer any questions that make you feel uncomfortable.

3. If you agree to be in this study, you will first be screened to see if you qualify to be in the study. If you qualify, you will be given the opportunity to complete the survey.

If you have any questions or comments you may contact the research staff at sexisthequestion@emory.edu.

Please read the information below about the study, and indicate whether you agree to participate in the study.

( ) I have read the information below. I agree to participate in this study.

( ) I do not agree to participate in the survey.

Section A: Demographics

What is the highest level of education you completed?

( ) Never attended school

( ) Less than high school

( ) Some high school

( ) High school diploma or GED

( ) Some college, Associate's Degree, or Technical Degree

( ) College, post graduate or professional school

( ) I prefer not to answer

( ) Don't know

What was your household income last year from all sources before taxes? That is, the total amount of money earned and shared **by all people living in your household.**

( ) $0 to $19,999 annually ($0 to $1667 monthly)

( ) $20,000 to $39,999 annually ($1668 to $3333 monthly)

( ) $40,000 to $74,999 annually ($3334 to $6250 monthly)

( ) $75,000 or more annually ($6251 or more monthly)

( ) I prefer not to answer

( ) Don't know

Including yourself**, how many people depend on this income?**

____

What kind of health insurance or health care coverage do you currently have?

Health insurance--health plans people get through employment or purchased directly as well as government programs (like Medicare and Medicaid) that provide medical care or help pay medical bills.

Choose all that apply:

[ ] A private health plan (through an employer or purchased directly)

[ ] Medicaid or Medicare

[ ] Some other Medical Assistance program

[ ] TRICARE (CHAMPUS)

[ ] Veterans Administration coverage

[ ] Some other health care plan

[ ] I don't currently have any health insurance

[ ] I prefer not to answer

[ ] Don't know

In the past 12 months**, have you seen a doctor, nurse, or other health care provider about your own health?**

( ) No

( ) Yes

( ) I prefer not to answer

( ) Don't know

At any of those times you were seen by a doctor or health care provider, were you offered an HIV test? An HIV test checks whether someone has the virus that causes AIDS.

( ) No

( ) Yes

( ) I prefer not to answer

( ) Don't know

Do you consider yourself to be:

( ) Heterosexual or "Straight"

( ) Homosexual or Gay

( ) Bisexual

( ) I prefer not to answer

( ) Don't know

Outness

Have you ever **told anyone that you are attracted to or have sex with men?**

( ) No

( ) Yes

( ) I prefer not to answer

( ) Don't know

Who of the following people have you told that you are attracted to or have sex with men?

	**No**	**Yes**	**Does not apply**
Gay, lesbian, or bisexual friends	( )	( )	( )
Friends who are not gay, lesbian, or bisexual	( )	( )	( )
Family members	( )	( )	( )
Health care provider	( )	( )	( )
Employer	( )	( )	( )
Fellow employees	( )	( )	( )

Marriage

Have you ever been legally married?

( ) Yes

( ) No

Are you currently legally married?

( ) Yes

( ) No

What is the gender of the partner to whom you are legally married?

( ) Male

( ) Female

( ) Transgender (Male-to-Female)

( ) Transgender (Female-to-Male)

Section B: Sexual Behavior: Female Sex Partners

The next questions are about having sex with women**. For these questions, "having sex" means oral, vaginal, or anal sex. Oral sex means mouth on the vagina or penis; vaginal sex means penis in the vagina; and anal sex means penis in the anus (butt).**

In the past 12 months (since [*SURVEY DATE*] of 2014), what types of sex have you had with a woman? (Check all that apply.)

[ ] Oral sex

[ ] Vaginal sex

[ ] Anal sex

[ ] I have not had any type of sex with a woman in the past 12 months

[ ] Prefer not to answer

[ ] Don't know

Section B: Sexual Behavior: Female Sex Partners

These next questions are about the last time **you had oral, vaginal or anal sex with a** woman**.**

Was the woman you had sex with that last time a main partner (someone you felt committed to above anyone else) or a casual partner (someone you didn't feel committed to or don’t know very well)?

( ) Main sex partner

( ) Casual sex partner

( ) I prefer not to answer

( ) Don't know

When you had sex that last time, did you have either **vaginal or anal sex?**

( ) No

( ) Yes

( ) I prefer not to answer

( ) Don't know

The last time you had sex with a woman, did you have either vaginal or **anal sex** without **using a condom?**

( ) No

( ) Yes

( ) I prefer not to answer

( ) Don't know

The last time you had sex with this partner, did you know her HIV status?

( ) No

( ) Yes

( ) I prefer not to answer

What was her HIV status?

( ) HIV-negative

( ) HIV-positive

( ) Indeterminate

( ) I prefer not to answer

Section B: Sexual Behavior: Male Sex Partners

The next questions are about having sex with men**. For these questions, "having sex" means oral or anal sex. Oral sex means he put his mouth on your penis or you put your mouth on his penis. Anal sex means you put your penis in his anus (butt) or he put his penis in your anus (butt).**

How old were you the first **time you had** <u>oral sex</u> **(mouth on the penis) with a man?**

____

How old were you the first **time you had** <u>anal sex</u> **(penis in the butt) with a man?**

____

Do you consider yourself to be a top, bottom, or versatile?

( ) Top

( ) Bottom

( ) Versatile

( ) I prefer not to answer

( ) Don't know

In the past 12 months, what types of sex have you had with other men?

( ) Only oral sex

( ) Only anal sex

( ) Both oral and anal sex

( ) I have not had oral or anal sex with a man in the past 12 months

( ) Prefer not to answer

( ) Don't know

In the past 12 months**, since [*SURVEY DATE*] of 2014, with how many different men have you had oral or anal sex?**

____

In the past 12 months**, since [*SURVEY DATE*] of 2014, with how many different men have you had anal sex?**

____

In the past 12 months**, since [*SURVEY DATE*] of 2014, with how many different men have you had oral sex?**

____

Of the [*NUMBER OF ANAL OR ORAL PARTNERS*] men you had oral or anal sex with in the past 12 months, how many of them did you have anal sex with?

____

In the past 12 months, did you have anal sex without using a condom?

( ) No

( ) Yes

( ) I prefer not to answer

( ) Don't know

In the past 12 months, with how many of these [*NUMBER OF ANAL PARTNERS*] male anal sex partners did you have anal sex without **using a condom?**

____

In the past 12 months, with how many of these [*NUMBER OF ANAL PARTNERS*] male anal sex partners did you have anal sex without **using a condom?**

____

Section B: Sexual Behavior: Male Sex Partners (1 Partner)

In the past 12 months, this male partner was a:

( ) Main partner (someone you felt committed to above anyone else)

( ) Casual partner (someone you didn't feel committed to or don't know very well)

( ) I prefer not to answer

( ) Don't know

Did you know his HIV status?

( ) No

( ) Yes

( ) I prefer not to answer

What was his HIV status?

( ) HIV-negative

( ) HIV-positive

( ) Indeterminate

( ) I prefer not to answer

Section B: Sexual Behavior: Male Sex Partners (>1)

In the past 12 months, the [*NUMBER OF ANAL OR ORAL PARTNERS*] [*NUMBER OF ANAL PARTNERS*] [*NUMBER OF ORAL PARTNERS*] male partners you told us about were:

( ) Only main partners (you felt committed to above anyone else)

( ) Only casual partners (you didn't feel committed to or don't know very well)

( ) Both main and casual partners

( ) I prefer not to answer

( ) Don't know

In the past 12 months, did you have anal sex without using a condom with a man whose HIV status you did not know?

( ) No

( ) Yes

( ) I prefer not to answer

( ) Don't know

In the past 12 months, did you have anal sex without using a condom with a man who was HIV positive?

( ) No

( ) Yes

( ) I prefer not to answer

( ) Don't know

In the past 12 months, did you have anal sex without using a condom with a man who was HIV negative?

( ) No

( ) Yes

( ) I prefer not to answer

( ) Don't know

Section B: Sexual Behavior: Male Sex Partners (Last Sex)

In the next few screens, we're going to ask some questions about your most recent male sex partner--that is, the last guy you had sex with.

To make the questions easier to ask, we'd like you to enter in this partner's initials or a nickname that is not his real name. If you prefer to leave his initials blank, we will refer to him as "your last partner".

What are the initials of or a nickname for your last male sex partner?

____

When was the last time you had either oral or anal sex with [*INITIALS / NICKNAME*]?

Month:

( ) January

( ) February

( ) March

( ) April

( ) May

( ) June

( ) July

( ) August

( ) September

( ) October

( ) November

( ) December

Year:: ____

Was [*INITIALS / NICKNAME*] a main partner (someone you felt committed to above anyone else) or a casual partner (someone you didn't feel committed to or don't know very well)?

( ) Main sex partner

( ) Casual sex partner

( ) I prefer not to answer

( ) Don't know

That last time you had sex with [*INITIALS / NICKNAME*], did you have receptive anal sex where he put his penis in your anus (you were the bottom)?

( ) No

( ) Yes

( ) I prefer not to answer

( ) Don't know

During that last time you had receptive anal sex, did [*INITIALS / NICKNAME*] use a condom?

( ) No

( ) Yes, but not the whole time

( ) Yes, the whole time

( ) I prefer not to answer

( ) Don't know

When you had sex that last time, did you have insertive anal sex where you put your penis in his anus (you were the top)?

( ) No

( ) Yes

( ) I prefer not to answer

( ) Don't know

During insertive anal sex that last time, did you use a condom?

( ) No

( ) Yes, but not the whole time

( ) Yes, the whole time

( ) I prefer not to answer

( ) Don't know

The last time you had sex with [*INITIALS / NICKNAME*], did you know his HIV status?

( ) No

( ) Yes

( ) I prefer not to answer

What was [*INITIALS / NICKNAME*]'s HIV status?

( ) HIV-negative

( ) HIV-positive

( ) Indeterminate

( ) I prefer not to answer

Group C

What was [*INITIALS / NICKNAME*]'s age?

( ) 19 years or younger

( ) 20 to 24 years

( ) 25 to 29 years

( ) 30 to 34 years

( ) 35 to 39 years

( ) 40 to 44 years

( ) 45 to 49 years

( ) 50 to 54 years

( ) 55 to 59 years

( ) 60 to 64 years

( ) 65 to 69 years

( ) 70 to 74 years

( ) 75 to 79 years

( ) 80 years or older

( ) Don't know

( ) Prefer not to answer

Was [*INITIALS / NICKNAME*] younger than you, older than you, or the same age as you?

[ ] Younger

[ ] Older

[ ] Same age

[ ] I prefer not to answer

[ ] Don't know

Which of the following best describes [*INITIALS / NICKNAME*]'s ethnic background?

[ ] American Indian or Alaska Native

[ ] Asian

[ ] Black or African American

[ ] Hispanic or Latino

[ ] Native Hawaiian or other Pacific Islander

[ ] White

[ ] I prefer not to answer

[ ] Don't know

Did you have sex with [*INITIALS / NICKNAME*] one time ('one night stand'), or more than one time?

( ) One time

( ) More than one time

( ) I prefer not to answer

( ) Don't know

How long have you been having a sexual relationship with [*INITIALS / NICKNAME*]?

.: ____

( ) Days

( ) Months

( ) Years

Do you expect to have sex with [*INITIALS / NICKNAME*] again?

( ) Yes

( ) No

( ) Don't know

( ) I prefer not to answer

As far as you know, during the time you were having a sexual relationship with [*INITIALS / NICKNAME*] , did he **have sex with other people?**

Would you say he:

( ) Definitely did not

( ) Probably did not

( ) Probably did

( ) Definitely did

( ) I prefer not to answer

( ) Don't know

During the time you were having a sexual relationship with [*INITIALS / NICKNAME*], did you **have sex with other people?**

( ) No

( ) Yes

( ) I prefer not to answer

( ) Don't know

Where did you first meet [*INITIALS / NICKNAME*]?

( ) Work

( ) School

( ) House party

( ) Mobile app (such as Grindr, Jackd, or Scruff)

( ) Internet

( ) Bar/Club

( ) Circuit party or rave

( ) Public sex environment (such as a bathhouse, sex club, sex resort, cruising area, private sex party, or adult bookstore)

( ) Place or worship (such as a church, synagogue, mosque)

( ) Other: ____

( ) I prefer not to answer

( ) Don't know

In the past 12 months, have you exchanged things like money or drugs for sex? Check all that apply.

[ ] No

[ ] Yes, I gave a sex partner things like drugs or money for sex

[ ] Yes, a sex partner gave me things like drugs or money for sex

[ ] I prefer not to answer

[ ] Don't know

In the past 12 months**, how often have you gone to a place where gay men hangout, meet or socialize? These could include bars, clubs, social organizations, parks, gay businesses, bookstores, sex clubs, etc.**

( ) More than once a day

( ) Once a day

( ) More than once a week

( ) Once a week

( ) More than once a month

( ) Once a month

( ) Less than once a month

( ) Never

( ) I prefer not to answer

( ) Don't know

In the past 12 months**, how often have you used the internet to** meet or socialize with gay men? **This includes visiting social network websites (such as Facebook or Myspace), websites directed towards gay men (such as Manhunt or Gay.com), dating websites, or the use of mobile social applications (such as Foursquare or Grindr).**

( ) More than once a day

( ) Once a day

( ) More than once a week

( ) Once a week

( ) More than once a month

( ) Once a month

( ) Less than once a month

( ) Never

( ) I prefer not to answer

( ) Don't know

How satisfied are you with your current sex life?

( ) Very satisfied

( ) Satisfied

( ) Unsure

( ) Dissatisfied

( ) Very Dissatisfied

( ) Don't know

( ) I prefer not to answer

Section C: Substance Use: Injection Drug Use

The next screens are about injection drug use. This means injecting drugs yourself or having someone who isn't a health care provider inject you.

Have you ever **in your life shot up or injected any drugs other than those prescribed for you? By shooting up, we mean anytime you might have used drugs with a needle, either by mainlining, skin popping, or muscling.**

( ) No

( ) Yes

( ) I prefer not to answer

( ) Don't know

In the past 12 months**, on average, how often did you inject?**

( ) More than once a day

( ) Once a day

( ) More than once a week

( ) Once a week

( ) More than once a month

( ) Once a month

( ) Less than once a month

( ) Never

( ) I prefer not to answer

( ) Don't know

Which drug do you inject most often?

( ) Speedball - Heroin and cocaine together

( ) Heroin, by itself

( ) Cocaine, by itself

( ) Crack

( ) Crystal, meth, tina, crank, ice

( ) Something else (Specify)

( ) I prefer not to answer

( ) Don't know

Section C: Substance Use: Non-Injection Drug Use

The next questions are about drugs that you may have used but did not inject.

In the past 12 months**, have you used any non-injection drugs (drugs you did** not **inject), other than those prescribed for you.**

( ) No

( ) Yes

( ) I prefer not to answer

( ) Don't know

In the past 12 months, which drugs did you use? (Check all that apply.)

[ ] Marijuana

[ ] Powdered cocaine (smoked or snorted)

[ ] Poppers (amyl nitrate)

[ ] X or Ecstasy

[ ] Painkillers (Oxycontin, Vicodin, Percocet)

[ ] Downers (Valium, Ativan, Xanax)

[ ] Crystal meth (tina, crank, ice)

[ ] Hallucinogens (LSD, mushrooms)

[ ] Special K (ketamine)

[ ] GHB

[ ] Crack cocaine

[ ] Other drug: ____

[ ] I prefer not to answer

[ ] Don't know

Drug use frequency

In the past 12 months, how often did you use [*NON-INJECTION DRUG*]?

( ) More than once a day

( ) Once a day

( ) More than once a week

( ) Once a week

( ) More than once a month

( ) Once a month

( ) Less than once a month

( ) I prefer not to answer

( ) Don't know

Alcohol and Drugs at last sex

Before or during the last time **you had sex with [*INITIALS / NICKNAME*], did you use:**

( ) Alcohol

( ) Drugs

( ) Both alcohol and drugs

( ) Neither one

( ) I prefer not to answer

( ) Don't know

For the next questions, a drink of alcohol is a 12 oz beer, a 5 oz glass of wine, or a 1.5 oz. shot of liquor. A 40 oz beer would count as 3 drinks. A cocktail with 2 shots would count as 2 drinks.

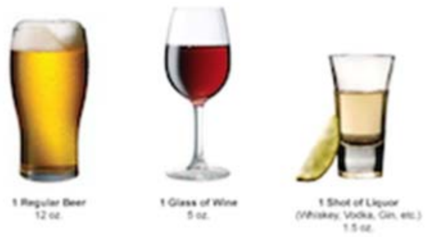

Before or during sex the last time you had sex with [*INITIALS / NICKNAME*], how many alcoholic drinks did you have?

____

The last time you had sex with [*INITIALS / NICKNAME*], which drugs did you use? Check all that apply.

[ ] Other drug: ____

[ ] I prefer not to answer

[ ] Don't know

Have you ever been tested for HIV? An HIV test checks whether someone has the virus that causes AIDS.

( ) No

( ) Yes

( ) I prefer not to answer

( ) Don't know

In the past 2 years, that is since [SURVEY DATE] of 2013, how many times have you been tested for HIV?

____

When did you have your most recent HIV test?

Month:

( ) January

( ) February

( ) March

( ) April

( ) May

( ) June

( ) July

( ) August

( ) September

( ) October

( ) November

( ) December

( ) Don't know

( ) Prefer not to answer

Year:: ____

Have you had an HIV test in the past 12 months (since [SURVEY DATE] of 2014)?

( ) Yes

( ) No

( ) Don't know

( ) I prefer not to answer

When you got tested in [*MONTH / YEAR OF HIV TEST*], where did you get tested?

( ) Private doctor's office

( ) HIV counseling and testing site

( ) Public health clinic/community health clinic

( ) Street outreach program/mobile unit

( ) Sexually transmitted disease clinic

( ) Hospital (inpatient)

( ) Correctional facility (jail or prison)

( ) Emergency room

( ) At home

( ) Other

( ) I prefer not to answer

( ) Don't know

Section D: At-home HIV Testing

A home HIV test is one that you can use to test yourself for HIV at home or another private location.

Have you ever heard about home HIV tests?

( ) No

( ) Yes

( ) I prefer not to answer

Have you ever used a home HIV test?

( ) No

( ) Yes

( ) I prefer not to answer

In the past 12 months, how many times have you tested yourself with a rapid home HIV test?

____

Are you interested in receiving a free home HIV test kit?

( ) Yes

( ) No

( ) Don't know

( ) I prefer not to answer

In the past 12 months, which of these home HIV tests have you used? Check all that apply.

[ ] Home Access HIV- Test System (where you prick your finger, colelct a blood sample on a card, and mail that card to a lab for testing)

[ ] Oraquick In-Home HIV Test (where you collect your own oral fluid sample by swabbing your mouth, use the testing device yourself, and read the test results within 20 minutes)

[ ] Other rapid HIV test (please specify): ____

[ ] Don't know

[ ] I prefer not to answer

You mentioned you used [*HOME HIV TEST*] to test yourself for HIV. Where did you get the home HIV test(s)? Check all that apply.

( ) Bought Online

( ) From a pharmacy

( ) From a friend

( ) From a sex partner

( ) From my doctor’s office

( ) From an HIV counseling and testing site

( ) From a Health Department

( ) From another research study

( ) Other location or person (Specify (do not include someone’s name)):
____

( ) I prefer not to answer

HIV Status

What was the result of your most recent HIV test?

( ) Negative

( ) Positive

( ) Never obtained results

( ) Indeterminate

( ) I prefer not to answer

( ) Don't know

Before your test in [*MONTH / YEAR OF HIV TEST*], did you ever test positive for HIV?

( ) No

( ) Yes

( ) I prefer not to answer

( ) Don't know

Was your test in [*MONTH / YEAR OF HIV TEST*] your first positive test?

( ) No

( ) Yes

( ) I prefer not to answer

( ) Don't know

When did you first test positive?

Month:

( ) January

( ) February

( ) March

( ) April

( ) May

( ) June

( ) July

( ) August

( ) September

( ) October

( ) November

( ) December

Year:: ____

Are you currently taking antiretroviral medicines to treat your HIV infection?

( ) No

( ) Yes

( ) I prefer not to answer

( ) Don't know

What is the main **reason you are not currently taking any antiretroviral medicines?**

( ) Not currently going to a health care provider for my HIV infection

( ) CD4 count and viral load are good

( ) Don't have money or insurance for antiretroviral medicines

( ) Don't want to take antiretroviral medicines

( ) Other

( ) I prefer not to answer

( ) Don't know

Serosorting

Which of the following strategies have you used in the past 12 months specifically to reduce your risk of getting HIV? Check all that apply.

[ ] I only had sex with HIV negative men

[ ] I avoided having sex with HIV positive men or men whose HIV status I did not know

[ ] I used condoms for anal sex for all my partners

[ ] I used condoms for anal sex if my partner was HIV positive or if I didn’t know his status

[ ] I was a top for all my partners

[ ] I was a top if my partner was HIV positive or if I didn’t know his status

[ ] I only had oral sex or masturbated my partner if he was HIV positive or I didn’t know his status

[ ] I did not have anal sex

[ ] None of the above

Which of the following strategies have you used in the past 12 months specifically to reduce the possibility of giving HIV to someone else? Check all that apply.

[ ] I only had sex with HIV positive men

[ ] I avoided having sex with HIV negative men or men whose HIV status I did not know

[ ] I used condoms for anal sex for all my partners

[ ] I used condoms for anal sex if my partner was HIV negative or if I didn’t know his status

[ ] I was a bottom for all my partners

[ ] I was a bottom if my partner was HIV negative or if I didn’t know his status

[ ] I did not have anal sex

[ ] None of the above

PrEP Module 1

Researchers are studying whether antiretroviral medicines could possibly be taken to prevent HIV.

Researchers are studying whether anti-HIV medicine (also called antiretrovirals)--a pill-- could possibly be taken to prevent HIV infection.

Before today, have you ever heard of people who do not have HIV taking antiretroviral medicines or anti-HIV medicines, a pill, to keep from getting HIV?

( ) No

( ) Yes

( ) I prefer not to answer

( ) Don't know

In the past 12 months (since [*SURVEY DATE*] of 2013), have you given your antiretroviral medicines to a sex partner who was HIV-negative because you thought it might keep them from getting HIV?

( ) No

( ) Yes

( ) I prefer not to answer

( ) Don't know

In the past 12 months, have you taken anti-HIV medicines after **sex because you thought it would keep you from getting HIV?**

( ) No

( ) Yes

( ) I prefer not to answer

( ) Don't know

In the past 12 months, have you taken anti-HIV medicines before **sex because you thought it would keep you from getting HIV?**

( ) No

( ) Yes

( ) I prefer not to answer

( ) Don't know

Did you get any of the anti-HIV medicines you took from the following people or places?

	**No**	**Yes**	**Prefer not to answer**	**Don't know**
Doctor or other health care provider	( )	( )	( )	( )
Sex partner, friend, relative, or acquaintance	( )	( )	( )	( )
Internet	( )	( )	( )	( )
Some other place	( )	( )	( )	( )

Would you be willing to take anti-HIV medicines every day **to lower your chances of getting HIV?**

( ) No

( ) Yes

( ) I prefer not to answer

( ) Don't know

PrEP Module 2

Pre-exposure prophylaxis (PrEP) is taking an antiretroviral pill, also called Truvada, every day for months or years to reduce a persons chance of getting HIV.

Before today, have you ever heard of people who do not have HIV taking PrEP, the antiretroviral medicine taken every day for months or years to reduce the risk of getting HIV?

( ) No

( ) Yes

In the past 12 months, have you had a discussion with a health care provider about taking PrEP?

( ) No

( ) Yes

( ) Don't know

( ) I prefer not to answer

In the past 12 months, when you discussed taking PrEP with a health care provider, did you receive the medicine or a prescription for the medicine?

( ) No

( ) Yes

( ) Don't know

( ) I prefer not to answer

In the past 12 months, have you taken PrEP to reduce the risk of getting HIV? Please remember that PrEP is the taking of pills every day for at least 2 months. Do not include times when you may have taken antiretroviral medicine for only a few days at a time.

( ) No

( ) Yes

( ) Don't know

( ) Prefer not to answer

Would you be willing to take anti-HIV medicines every day **to lower your chances of getting HIV?**

( ) No

( ) Yes

( ) I prefer not to answer

( ) Don't know

PEP

Post-exposure prophylaxis (PEP) is taking antiretroviral pills for 28 days after a person has had a single high-risk exposure to HIV.

In the past 12 months, have you taken PEP to reduce the risk of getting HIV?

( ) No

( ) Yes

( ) Don't know

( ) I prefer not to answer

Acute Infection

Now we would like to ask you a few questions about your understanding of HIV infection.

Do you think that after a person is infected with HIV, they can show symptoms within a month of being infected?

( ) No

( ) Yes

( ) Don't know

How long after the exposure to infection do you think these symptoms occur?

( ) 1 Day

( ) 3 Days

( ) 1 Week

( ) 2-4 Weeks

( ) Don't know

How long do you think these symptoms last?

( ) 1 Day

( ) 3 Days

( ) 1 Week

( ) 2-4 Weeks

( ) Longer than 4 weeks

( ) Don't know

About what percent of people who are infected with HIV show symptoms shortly after they are infected?

0 ________________________[__]______________________________ 100

Which of the following do you think are symptoms people have shortly after HIV infection?

[ ] Fever

[ ] Fatigue (feeling tired)

[ ] Muscle or joint soreness

[ ] Headache

[ ] Excessive thirst

[ ] Frequent urination

[ ] Weight loss

[ ] Sweaty hands or feet

[ ] Don't know

Have you ever had any disease symptoms and worried that you had recently (in the prior month) become infected with HIV?

( ) No

( ) Yes

Viral STI Diagnoses

Has a doctor, nurse or other health care provider ever **told you that you had any of the following? Check all that apply.**

[ ] Hepatitis

[ ] Genital herpes

[ ] Genital warts

[ ] Human papillomavirus or HPV

[ ] None of the above

[ ] I prefer not to answer

[ ] Don't know

What type or types of hepatitis have you had? Check all that apply.

[ ] Hepatitis A

[ ] Hepatitis B

[ ] Hepatitis C

[ ] Other

[ ] I prefer not to answer

[ ] Don't know

There are vaccines or shots that can prevent some types of hepatitis. Have you ever **had a hepatitis vaccine?**

( ) No

( ) Yes

( ) I prefer not to answer

( ) Don't know

What type or types of hepatitis vaccine have you had?

( ) Hepatitis A vaccine

( ) Hepatitis B vaccine

( ) Both Hepatitis A and B vaccine

( ) I prefer not to answer

( ) Don't know

Bacterial STI Diagnoses

In the past 12 months **(since [*SURVEY DATE*] or 2013), has a doctor, nurse or other health care provider told you that you had any of the following? Check all that apply.**

[ ] Gonorrhea

[ ] Chlamydia

[ ] Syphilis

[ ] None of the above

[ ] I prefer not to answer

[ ] Don't know

Bacterial STI Testing

Even though a doctor, nurse, or other health care provider did not tell you that you had Gonorrhea**, in the past 12 months (since [*SURVEY DATE*] of 2013) were you tested for** Gonorrhea**?**

( ) No

( ) Yes

( ) I prefer not to answer

( ) Don't know

Even though a doctor, nurse, or other health care provider did not tell you that you had chlamydia**, in the past 12 months (since [*SURVEY DATE*] of 2013) were you tested for** chlamydia**?**

( ) No

( ) Yes

( ) I prefer not to answer

( ) Don't know

Even though a doctor, nurse, or other health care provider did not tell you that you had syphilis**, in the past 12 months (since [*SURVEY DATE*] of 2013) were you tested for** syphilis**?**

( ) No

( ) Yes

( ) I prefer not to answer

( ) Don't know

HPV

A vaccine to prevent human papillomavirus (HPV) infection is available and is called the HPV shot, cervical cancer vaccine, GARDASIL®, or CERVARIX®. Have you ever received the HPV vaccine?

( ) No

( ) Yes

( ) I prefer not to answer

( ) Don't know

How old were you when you received your first dose of the HPV vaccine?

____

Section F. Assessment of Prevention Activities

In the past 12 months**, have you gotten any free condoms, not counting those given to you by a friend, relative, or sex partner?**

( ) No

( ) Yes

( ) I prefer not to answer

( ) Don't know

In the past 12 months**, have you had a one-on-one conversation with an outreach worker, counselor, or prevention program worker about ways to prevent HIV? Don't count the times where you had a conversation as part of an HIV test.**

( ) No

( ) Yes

( ) I prefer not to answer

( ) Don't know

In the past 12 months**, have you been a participant in any organized session(s) involving a small group of people to discuss ways to prevent HIV? Don't include discussions you had with a group of friends.**

( ) No

( ) Yes

( ) I prefer not to know

( ) Don't know

Conversations with Main Partners

In the past 12 months**, how often have you had one-on-one conversations** with a main sexual partner **about ways to prevent HIV?**

( ) Never

( ) Rarely

( ) Sometimes

( ) Often

( ) Very Often

( ) I prefer not to answer

( ) Don't Know

In the past 12 months, how often have you discussed the following topics with your main male sexual partner?

	**Very often**	**Often**	**Sometimes**	**Rarely**	**Never**	**Prefer not to answer**
Your last HIV test and test results	( )	( )	( )	( )	( )	( )
Your partner's last HIV test and test results	( )	( )	( )	( )	( )	( )
Getting tested for HIV/Getting tested for HIV together	( )	( )	( )	( )	( )	( )
Using condoms	( )	( )	( )	( )	( )	( )
Taking medicines to treat HIV infection (e.g., antiretroviral therapy or ART)	( )	( )	( )	( )	( )	( )

Your or your partner's viral load	( )	( )	( )	( )	( )	( )
Taking HIV medicines daily to reduce the chance of being HIV infected (e.g., pre-exposure prophylaxis, PrEP)	( )	( )	( )	( )	( )	( )
Taking HIV medicines after sex to reduce the chance of being HIV infected (e.g., post-exposure prophylaxis, nPEP)	( )	( )	( )	( )	( )	( )
Which sexual roles or positions (i.e., insertive/top or receptive/bottom) you would engage in based on your HIV status or your partner's HIV status	( )	( )	( )	( )	( )	( )
Which sexual activities (i.e., oral and anal intercourse) you would engage in based on your HIV status or your partner's HIV status	( )	( )	( )	( )	( )	( )
Making agreements with your main male sexual partner about having sex with other partners (i.e., whether to, when, with whom, and what kind of	( )	( )	( )	( )	( )	( )

sex that takes place inside and outside the relationship)						

Conversations with Casual Partners

In the past 12 months**, how often have you had one-on-one conversations** with a casual sexual partner **about ways to prevent HIV?**

( ) Never

( ) Rarely

( ) Sometimes

( ) Often

( ) Very Often

( ) I prefer not to answer

( ) Don't know

In the past 12 months, how often have you discussed **the following topics with your** *casual male sexual partners***?**

	**Very often**	**Often**	**Sometimes**	**Rarely**	**Never**	**Prefer not to answer**
Your last HIV test and test results	( )	( )	( )	( )	( )	( )
Your partner's last HIV test and test results	( )	( )	( )	( )	( )	( )
Getting tested for HIV/Getting tested for HIV together	( )	( )	( )	( )	( )	( )
Using condoms	( )	( )	( )	( )	( )	( )
Taking medicines to treat HIV infection (e.g., antiretroviral therapy or ART)	( )	( )	( )	( )	( )	( )

Your or your partner's viral load	( )	( )	( )	( )	( )	( )
Taking HIV medicines daily to reduce the chance of being HIV infected (e.g., pre-exposure prophylaxis, PrEP)	( )	( )	( )	( )	( )	( )
Taking HIV medicines after sex to reduce the chance of being HIV infected (e.g., post-exposure prophylaxis, nPEP)	( )	( )	( )	( )	( )	( )
Which sexual roles or positions (i.e., insertive/top or receptive/bottom) you would engage in based on your HIV status or your partner's HIV status	( )	( )	( )	( )	( )	( )
Which sexual activities (i.e., oral and anal intercourse) you would engage in based on your HIV status or your partner's HIV status	( )	( )	( )	( )	( )	( )
Making agreements with your main male sexual partner about having sex with other partners (i.e., whether to, when, with whom, and what kind of	( )	( )	( )	( )	( )	( )

sex that takes place inside and outside the relationship)						

HIV Testing Intentions and Perceptions

How sure are you that you could get an HIV test within the next 3-6 months **if you wanted to?**

( ) Very sure I could not

( ) Somewhat sure I could not

( ) Slightly sure I could

( ) Somewhat sure I could

( ) Very sure I could

( ) I prefer not to answer

( ) Refuse to answer

How likely is it that you will get an HIV test within the next 3-6 months?

( ) Very unlikely

( ) Somewhat unlikely

( ) Slightly likely

( ) Somewhat likely

( ) Very likely

( ) I'd prefer not to answer

( ) Refuse to answer

Most gay men I know get tested for HIV at least every 3-6 months

( ) Strongly agree

( ) Agree

( ) Neither agree nor disagree

( ) Disagree

( ) Strongly disagree

( ) I prefer not to answer

( ) Don't know

Prevention Campaigns

In the past 12 months, how often did you see or hear the following slogans or messages?

Let's stop HIV together:

( ) Never

( ) Rarely

( ) Sometimes

( ) Often

( ) Very Often

( ) I prefer not to answer

( ) Don't know

On a scale of 0 to 5, where 0 means "not very effective" and 5 means "very effective", how effective do you think this slogan or message is?

( ) 0 (Not Effective)

( ) 1

( ) 2

( ) 3

( ) 4

( ) 5 (Very Effective)

In the past 12 months, how often did you see or hear the following slogans or messages?

Protest:

( ) Never

( ) Rarely

( ) Sometimes

( ) Often

( ) Very Often

( ) I prefer not to answer

( ) Don't know

On a scale of 0 to 5, where 0 means "not very effective" and 5 means "very effective", how effective do you think this slogan or message is?

( ) 0 (Not Effective)

( ) 1

( ) 2

( ) 3

( ) 4

( ) 5 (Very Effective)

In the past 12 months, how often did you see or hear the following slogans or messages?

ACT Against AIDS:

( ) Never

( ) Rarely

( ) Sometimes

( ) Often

( ) Very Often

( ) I prefer not to answer

( ) Don't know

On a scale of 0 to 5, where 0 means "not very effective" and 5 means "very effective", how effective do you think this slogan or message is?

( ) 0 (Not Effective)

( ) 1

( ) 2

( ) 3

( ) 4

( ) 5 (Very Effective)

In the past 12 months, how often did you see or hear the following slogans or messages?

WE > AIDS
greaterthan.org

Greater than AIDS:

( ) Never

( ) Rarely

( ) Sometimes

( ) Often

( ) Very Often

( ) I prefer not to answer

( ) Don't know

On a scale of 0 to 5, where 0 means "not very effective" and 5 means "very effective", how effective do you think this slogan or message is?

( ) 0 (Not Effective)

( ) 1

( ) 2

( ) 3

( ) 4

( ) 5 (Very Effective)

In the past 12 months, how often did you see or hear the following slogans or messages?

Start Talking. Stop HIV.

( ) Never

( ) Rarely

( ) Sometimes

( ) Often

( ) Very Often

( ) I prefer not to answer

( ) Don't know

On a scale of 0 to 5, where 0 means "not very effective" and 5 means "very effective", how effective do you think this slogan or message is?

( ) 0 (Not Effective)

( ) 1

( ) 2

( ) 3

( ) 4

( ) 5 (Very Effective)

In the past 12 months, how often did you see or hear the following slogans or messages?

Testing Makes us Stronger:

( ) Never

( ) Rarely

( ) Sometimes

( ) Often

( ) Very Often

( ) I prefer not to answer

( ) Don't know

On a scale of 0 to 5, where 0 means "not very effective" and 5 means "very effective", how effective do you think this slogan or message is?

( ) 0 (Not Effective)

( ) 1

( ) 2

( ) 3

( ) 4

( ) 5 (Very Effective)

In the past 12 months, how often did you see or hear the following slogans or messages?

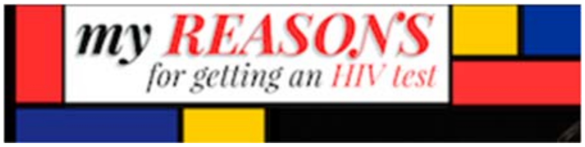

Reasons:

( ) Never

( ) Rarely

( ) Sometimes

( ) Often

( ) Very Often

( ) I prefer not to answer

( ) Don't know

On a scale of 0 to 5, where 0 means "not very effective" and 5 means "very effective", how effective do you think this slogan or message is?

( ) 0 (Not Effective)

( ) 1

( ) 2

( ) 3

( ) 4

( ) 5 (Very Effective)

In the past 12 months, how often did you see or hear the following slogans or messages?

HIV Treatment Works:

( ) Never

( ) Rarely

( ) Sometimes

( ) Often

( ) Very Often

( ) I prefer not to answer

( ) Don't know

On a scale of 0 to 5, where 0 means "not very effective" and 5 means "very effective", how effective do you think this slogan or message is?

( ) 0 (Not Effective)

( ) 1

( ) 2

( ) 3

( ) 4

( ) 5 (Very Effective)

As far as you know, did you participate in the “Sex is the Question” Survey between December 2013 and May 2014?

( ) Yes

( ) No

( ) I'm not sure

For this national study, we are recruiting a large number of men like you. Can you tell us the name of social networking website where we could reach other men like you who might like to complete this survey?

____

Future Contact

The PRISM Health team conducts many research projects at Emory University. Would you like to be contacted for potential participation in our future projects?

( ) Yes

( ) No

Please provide the email address you would like for us to use to contact you for future studies.

____

Survey End

Thank you for taking our survey! Your response is very important to us!

If you have any questions or comments, you may contact study staff at sexisthequestion@emory.edu.

To get more information about HIV, please visit: www.cdc.gov/hiv

Otherwise, you can close your browser.
